# Right ventricular remodeling and dysfunction among the spectrum of heart failure phenotypes

**DOI:** 10.1186/1532-429X-18-S1-P73

**Published:** 2016-01-27

**Authors:** Harris Wang, Lingyu Xu, Kelvin Chow, Joseph J Pagano, Anna Schmidt, James A White, Evangelos Michelakis, Justin Ezekowitz, Jason Dyck, Mark Haykowsky, Gavin Y Oudit, Richard B Thompson, Ian Paterson

**Affiliations:** 1 Cardiology, University of Alberta, Edmonton, AB Canada; 2 Biomedical Engineering, University of Alberta, Edmonton, AB Canada; 3 University of Calgary, Calgary, AB Canada; 4 University of Alberta, Edmonton, AB Canada

## Background

Right ventricular dysfunction (RVD) and enlargement (RVE) are increasingly associated with poor outcomes however their prevalence among patients with heart failure and those at risk has not been well characterized.

## Methods

Healthy controls and patients with heart failure related structural heart disease underwent a standard cardiac magnetic resonance examination. Right and left ventricular ejection fraction (EF) and end diastolic volumes (EDV) were traced from cine imaging. RVD was defined as RVEF less than mean minus 2 standard deviations of healthy gender matched controls. RVE was defined as either indexed RVEDV *or* RVEDV/LVEDV exceeding mean plus 2 standard deviations of healthy gender matched controls. The prevalence of RV abnormalities and pattern of ventricular remodeling was compared among patient subgroups.

## Results

89 healthy controls, age 57 ± 10 years, and 502 patients, age 56 ± 16 years, were included. Among controls, RVEF was 61 ± 6% for women and 60 ± 5.6% for men, p = 0.5, indexed RVEDV was 63 ± 11 mL/m^2^ for women and 71 ± 12 mL/m^2^ for men, p <0.05, and mean RVEDV/LVEDV was 0.94 ± 0.12 for women and 0.98 ± 0.14 for men, p = 0.19. Among patients, RVD was detected in 47%, RVE in 38% and either RV abnormality in 61%. RV abnormalities were common in all patient subgroups, ranging from 31% in hypertensive heart disease to 100% in right sided valvular heart disease.

## Conclusions

RV dysfunction and/or enlargement are prevalent among patients with heart failure related structural heart disease. Routine assessment is therefore recommended in 3-D cardiac imaging.

**Figure 1:**
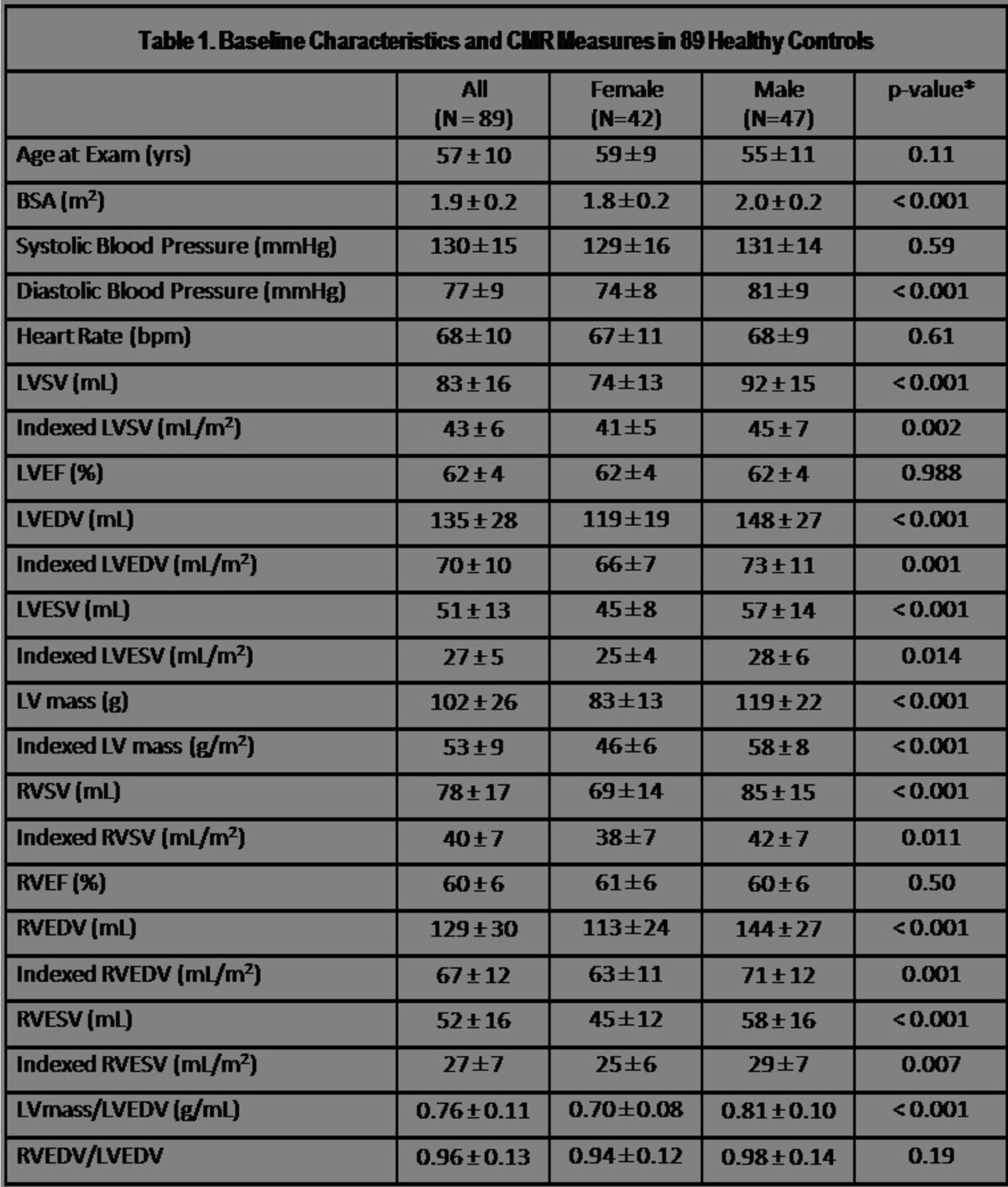
**All values are given as mean +/- standard deviation except for number of subjects**. * p-value of comparison between male and female healthy controls

**Figure 2:**
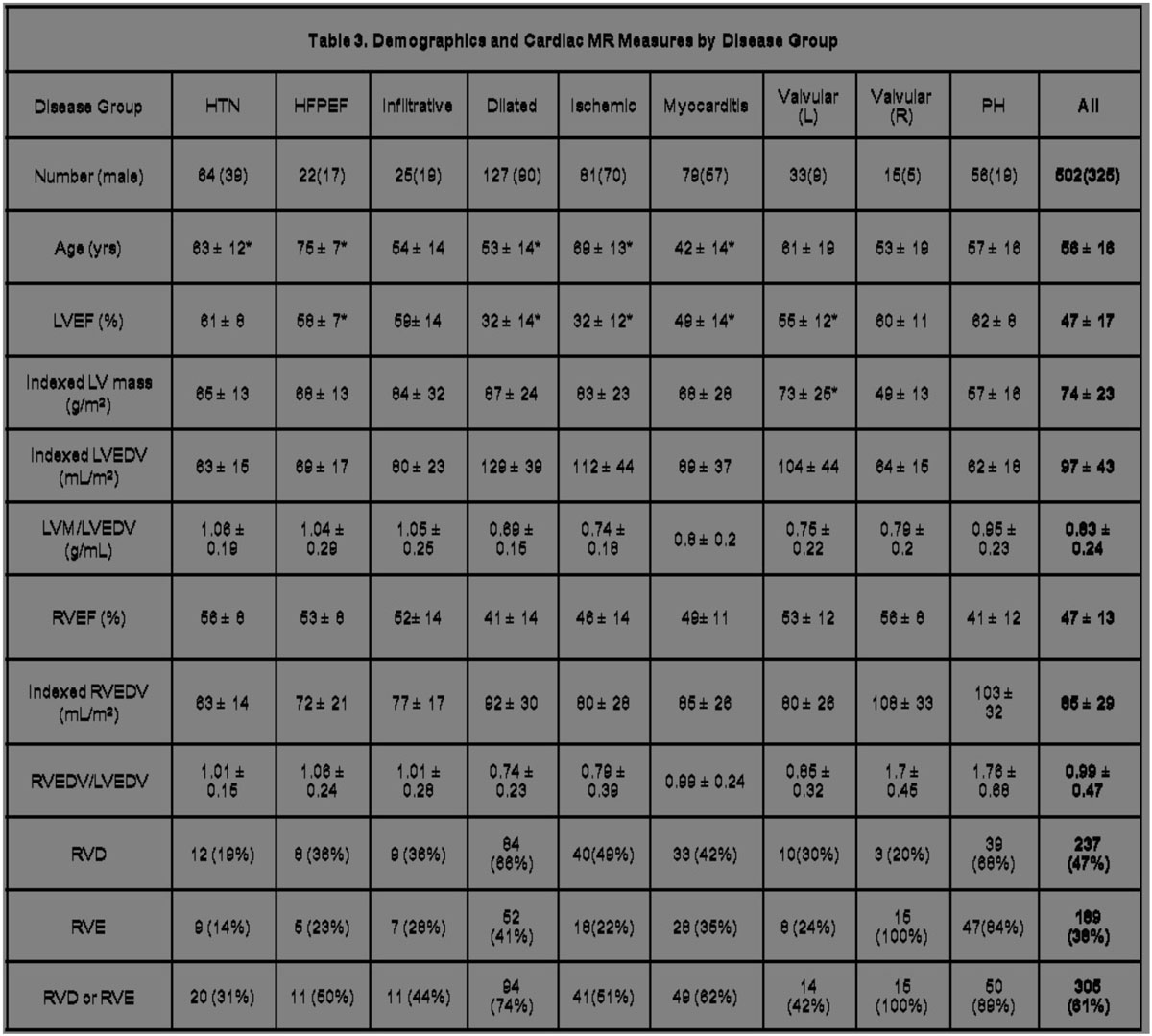
**All values are given as mean +/- standard deviation except for number of subjects**.

